# Supplementary figures and images for: Enriched Pathways of Calcium Regulation, Cellular/Oxidative Stress, Inflammation, and Cell Proliferation Characterize Gluteal Muscle of Standardbred Horses between Episodes of Recurrent Exertional Rhabdomyolysis
Source: Genes (Basel). 2022 Oct 14;13(10):1853. doi: 10.3390/genes13101853 (PMC9601720; doi:10.3390/genes13101853)

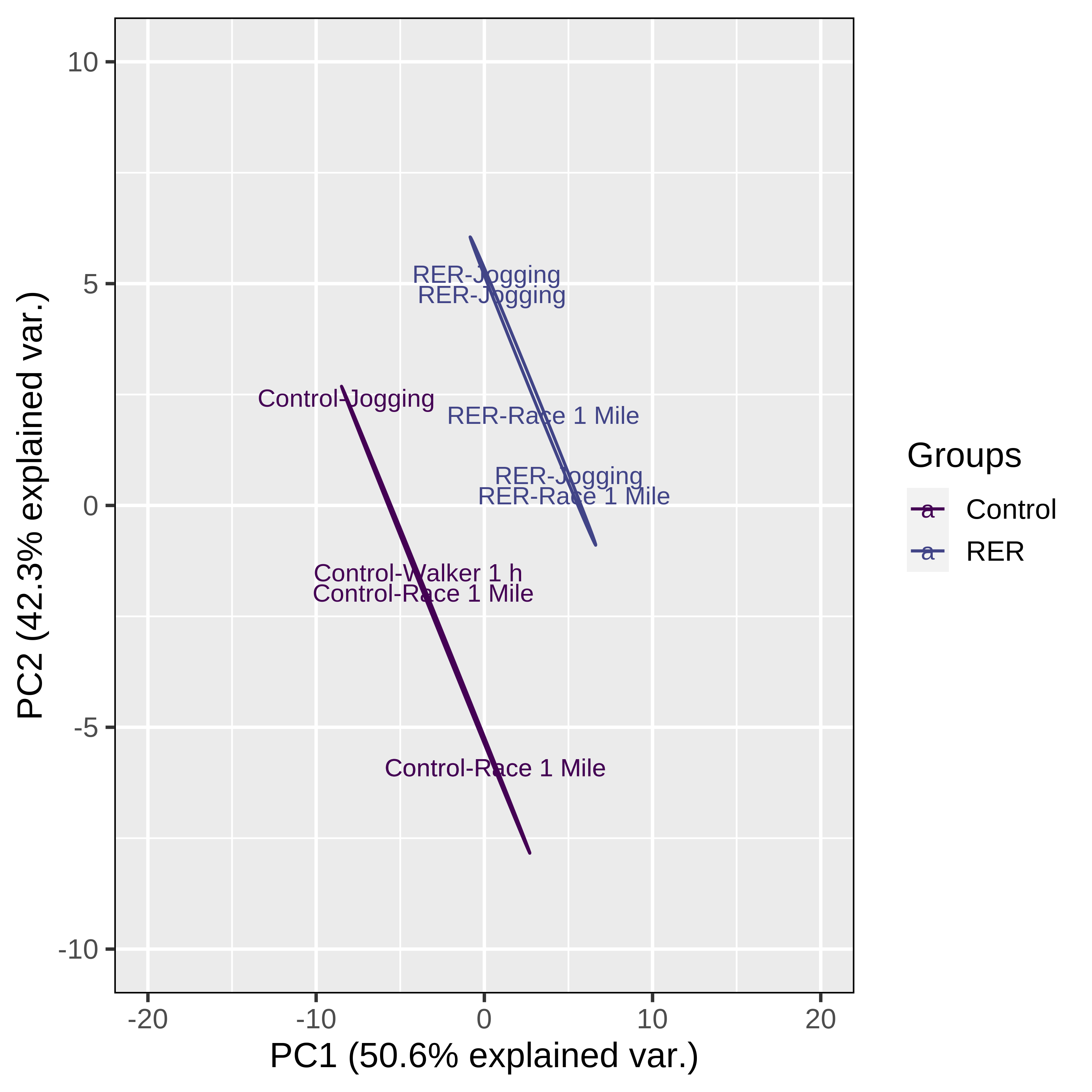

Supplement: Supplementary file 1 [file genes-13-01853-s001.zip › Archive/S2 ER_PCA_fitted.tiff]
